# Supplementary material for: Maintenance Therapy for Pancreatic Cancer, a New Approach Based on the Synergy between the Novel Agent GP-2250 (Misetionamide) and Gemcitabine
Source: Cancers (Basel). 2024 Jul 22;16(14):2612. doi: 10.3390/cancers16142612 (PMC11275110; doi:10.3390/cancers16142612)
Supplement: Supplementary file 1 [file cancers-16-02612-s001.zip › cancers-3124484-supplementary.pdf]

cancers

MDPI

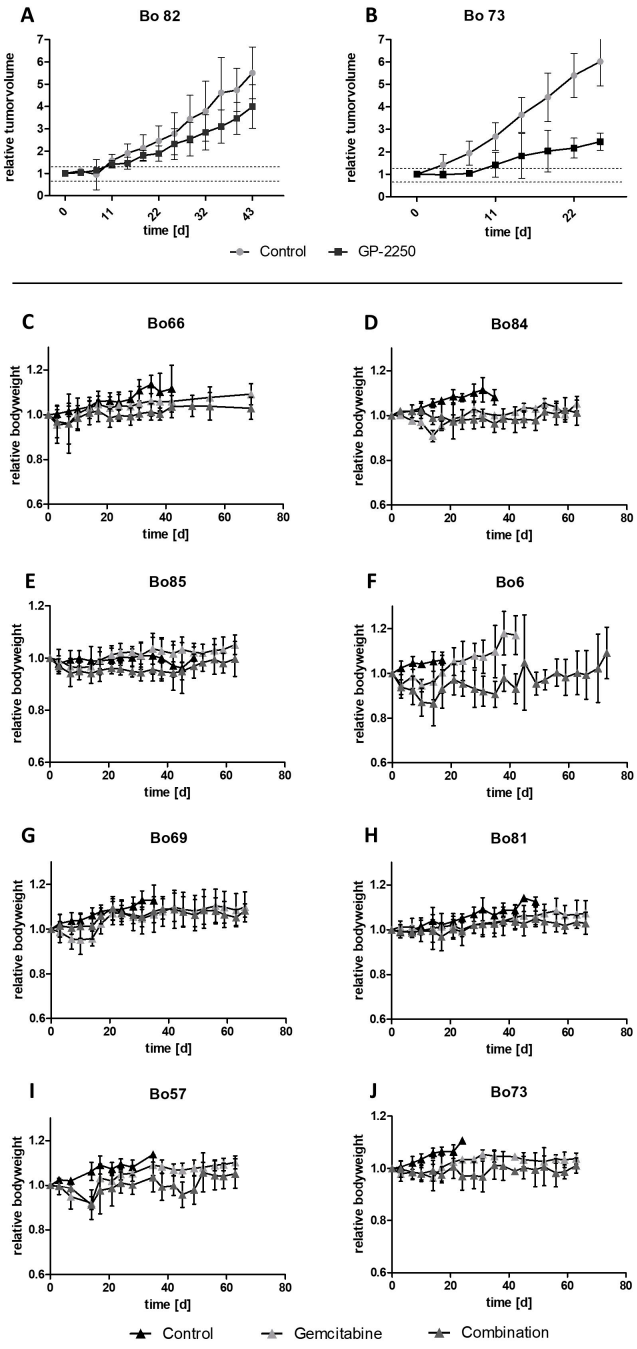

Supplementary Figure S1. (**A**,**B**) **Monotherapy in vivo.** Effects of 500 mg/kg*BW GP-2250 versus vehicle treated control on the subcutaneous tumor growth in nude mice in vivo. Nude mice with tumors of Bo82 (A), Bo73 (B) were treated with GP-2250 (500 mg/kg*BW) three times a week or vehicle (control) for up to 9 weeks. The tumor volume was measured twice weekly. (**C**–**J**) **Changes in body weight of mice undergoing maintenance therapy in vivo** with 500 mg/kg*BW GP-2250 in combination with Gemcitabine (50 mg/kg*BW) versus Gemcitabine alone after treatment with Gemcitabine (100 mg/kg*BW) in combination with nab-Paclitaxel (30 mg/kg*BW) for two weeks. Nude mice with tumors of B66 (C), Bo84 (D), Bo85 (E), Bo6 (F), Bo69 (G), Bo81 (H), Bo57 (I), and Bo73 (J) were incubated after initial therapy with Gemcitabine monotherapy (50 mg/kg*BW) twice weekly, GP-2250 (500 mg/kg*BW) three times a week in combination with Gemcitabine (50 mg/kg*BW) twice weekly or treated with vehicle (control) for up to 9 weeks. The asterisk symbols indicate differences between the combination treatment and the Gemcitabine monotherapy. *** $p \leq 0.001$, ** $p \leq 0.01$, * $p \leq 0.05$, n.s. $p > 0.05$ (one-way ANOVA followed by Tukey's post hoc test).
